# NGF Expression and Elevation in Hip Osteoarthritis Patients with Pain and Central Sensitization

**DOI:** 10.1155/2021/9212585

**Published:** 2021-09-18

**Authors:** Yoshihisa Ohashi, Kentaro Uchida, Kensuke Fukushima, Masashi Satoh, Tomohisa Koyama, Maho Tsuchiya, Hiroki Saito, Naonobu Takahira, Gen Inoue, Masashi Takaso

**Affiliations:** ^1^Department of Orthopaedic Surgery, Kitasato University School of Medicine, 1-15-1 Minami-ku, Kitasato, Sagamihara City, Kanagawa 252-0374, Japan; ^2^Department of Immunology, Kitasato University School of Medicine, 1-15-1 Minami-ku Kitasato, Sagamihara City, Kanagawa, Japan; ^3^Department of Rehabilitation, Kitasato University School of Allied Health Sciences, 1-15-1 Minami-ku Kitasato, Sagamihara City, Kanagawa, Japan

## Abstract

Osteoarthritis (OA) is a chronic degenerative musculoskeletal disease that causes articular cartilage degeneration and chronic pain. Research into OA animal models suggests that elevated NGF levels in the synovium contribute to pain and central sensitization (CS). However, it is unclear whether synovial NGF contributes to CS in patients with OA. We investigated the association between synovial NGF expression and clinical assessments of pain and CS in hip OA (hOA) patients. We also aimed to identify which cells in the synovium of hOA patients express NGF. Sixty-six patients who received total hip replacement and a diagnosis of hOA were enrolled. We measured *NGF* mRNA expression in synovial samples obtained from 50 patients using qPCR and analyzed the correlation of *NGF* expression with the CS inventory (CSI) score and Japanese Orthopaedic Association (JOA) score, a clinical scoring system for OA. To identify the synovial cells expressing NGF, we analyzed *NGF* mRNA expression in CD14+ and CD14- cells, which represent macrophage-rich and fibroblast-rich fractions, respectively, extracted from 8 patients. To further identify which macrophage subtypes express NGF, we examined *NGF* mRNA expression in CD14^high^ and CD14^low^ cells sorted from 8 patients. Synovial *NGF* mRNA expression was negatively correlated with JOA score but positively correlated with CSI score (JOA pain, *r* = −0.337, *P* = 0.017; CSI score, *r* = 0.358, *P* = 0.011). Significantly greater levels of *NGF* were observed in CD14- cells compared to CD14+ cells (*P* = 0.036) and in CD14^high^ cells compared to CD14^low^ cells (*P* = 0.008). In conclusion, synovial NGF expression is correlated with the degree of pain and CS in hOA patients. NGF is predominantly expressed in synovial fibroblasts. Further, CD14^high^ synovial macrophages expressed higher levels of NGF. Our results may provide a novel NGF-targeted therapeutic strategy for hOA pain.

## 1. Introduction

Osteoarthritis (OA) is a multifaceted disease that features continued cartilage degeneration, synovitis, osteophyte development, and subchondral bone remodeling, which lead to chronic pain. Evidence suggests that central sensitization (CS) is a factor of treatment-resistant chronic pain among individuals with OA [1, 2]. Further, individuals exhibiting pain and CS components are resistant to conservative treatment, including nonsteroidal anti-inflammatory drugs (NSAIDs) and other pain medication [3]. We previously reported that 5% of hip OA (hOA) patients have a CS component [4]. However, the mechanisms underlying CS in hOA are not well understood.

The neurotrophin nerve growth factor (NGF) modulates pain sensation [5] and has been shown to be elevated in individuals with chronic pain conditions, causing a heightened perception of pain [6, 7]. Synovial tissue is a major source of NGF in joints. Elevated levels of NGF have been observed in the synovial fluid and synovial tissue in patients with knee OA (kOA) [6]. Studies in experimental animal OA models have highlighted a pivotal role for elevated NGF in joint tissues in CS [7]. However, it is unclear whether elevated NGF expression in synovial tissue contributes to pain and CS in patients with hOA.

The synovium is mainly composed of synovial fibroblasts and macrophages. Under inflammatory conditions, synovial fibroblasts produce pain-related signals. Previous studies have shown that NGF is expressed in the synovium and is produced by synovial fibroblasts isolated from patients with kOA [8, 9]. However, NGF expression levels in synovial fibroblasts from patients with hOA have not been examined.

Interestingly, several human studies have suggested a correlation between synovial macrophages and pain. Activated macrophages are found in 76% of patients with symptomatic kOA [10]. Further, the presence of joint pain in the fingers, wrists, ankles, and big toes has been linked to the presence of activated macrophages in these joints. Immunohistochemical analysis has shown that NGF is expressed by specific macrophage populations in the synovium of kOA patients [11]. However, the osteoarthritic synovium comprises a heterogeneous population, and it remains unclear which subsets of macrophages in the synovium of hOA patients express NGF.

Here, we investigated the association between synovial NGF expression and clinical assessments for pain and CS in patients with hOA. We also aimed to identify which cell populations in the synovium of hOA patients express NGF.

## 2. Materials and Methods

### 2.1. Participants

Sixty-six patients who underwent total hip replacement (THR) at a single center and a diagnosis of hOA (Tonnis grade 2–3) were enrolled in the present study. Samples taken from 50 patients were used to investigate the correlation between synovial *NGF* mRNA expression and clinical scores. Eight patients were used to measure *NGF* mRNA expression in synovial macrophages and fibroblasts, and the remaining 8 patients were used to examine the cell subsets in the macrophage fraction and *NGF* mRNA expression within the population. Synovial samples were extracted from all operated hip joints during the THR procedure. This study received ethical approval from Kitasato University Institutional Review Board (reference number: B19-259). Written consent was obtained from all participants for the harvesting of their synovial tissue for use in this study.

### 2.2. Clinical Assessments

Radiographic assessment of hOA progression was determined according to the Tonnis classification system [12] as follows: 0, no changes; 1, mild narrowing of the joint space, mild lipping at the joint margin, and mild sclerosis of the femoral head or acetabulum; 2, appearance of small bony cysts, additional narrowing of the joint space, and modest loss of femoral head sphericity; 3, presence of large cysts, marked narrowing of the joint space, marked femoral head deformity, and avascular necrosis. While both the Tonnis classification and Kellgren-Lawrence classification are used to classify hip osteoarthritis severity, the Kellgren-Lawrence classification is widely used in the knee osteoarthritis literature, while the Tonnis classification is often used in the hip osteoarthritis literature. The Tonnis classification has similar definitions to the Kellgren-Lawrence classification but includes cysts as part of its definitions.

The CS inventory (CSI), a comprehensive self-report outcome measure, is comprised of 25 self-reported items on emotional and somatic symptoms [13–15]. Each item is scored on a 5-point scale (0, never; 1, rarely; 2, sometimes; 3, often; 4, always), with the total score, ranging from 0 to 100 points, indicating the degree of symptomatology. The CSI was established as a screening tool to help clinicians identify patients with central sensitivity syndrome, in which CS may be a root etiology. Previous studies have used the CSI to study the association of pain with CS components in patients with kOA and hOA [4, 16, 17]. In this study, we asked patients to complete the CSI to assess whether they exhibit CS components. We provided patients with an explanation of the CSI and left them to complete the CSI themselves one month before THR.

Additionally, we used the Japanese Orthopaedic Association (JOA) hip score to evaluate other clinical parameters. The JOA hip score is widely used in Japan to assess hip diseases and comprises 4 categories: pain (0–40 points), range of motion (ROM 0–20 points), gait (0–20 points), and performance of activities of daily living (0–20 points) [18]. The ROM score was determined based on hip flexion angle and abduction angle. Higher scores represent milder conditions.

These data were used to examine the correlation between synovial *NGF* mRNA expression and clinical symptoms.

### 2.3. Isolation of RNA and qPCR

Patients' synovial cells and synovial samples were subjected to RNA extraction using TRIzol (Invitrogen, Carlsbad, California, USA) based on manufacturer's protocol. The extracted RNA formed the template for synthesizing first-strand complementary DNA (cDNA) using SuperScript III RT (Invitrogen). Briefly, DNA was amplified in a reaction mixture containing TB green Premix Ex Taq (Takara, Kyoto, Japan), a specific primer set, and cDNA in a qPCR detection system (CFX96; Bio-Rad, Hercules, California, USA) using the following PCR program: denaturation at 95°C for 1 min, followed by 40 cycles at 95°C for 10 s and 60°C for 30 s. qPCR was used to determine mRNA expression of *NGF*, *CD14*, and *CD90* in synovial samples and cells from hOA patients. The sequences of the PCR primer pairs used are provided in Table 1. *NGF*, *CD14*, and *CD90* mRNA expression was normalized to that of *GAPDH*.

### 2.4. Isolation of Synovial Macrophages and Fibroblasts

Synovial samples collected from each hip joint during THR were digested with type I collagenase for 24 h at 37°C to isolate mononuclear cells. After reacting with 0.5 ml biotin-conjugated anti-CD14 human monoclonal antibody (BioLegend) for 30 min at 4°C, the cells were washed twice in phosphate-buffered saline (PBS) and reacted with streptavidin-conjugated magnetic beads (BD Biosciences). The cells were subsequently transferred to a cell separation system (IMag; BD Biosciences) and left to stand for 30 min at 4°C. After removal from the magnetic support, 5 ml of prewarmed (37°C) *α*-minimum essential medium (MEM) was added, and the cells were centrifuged at 280 g for 5 min to isolate CD14+ (macrophage-rich fraction) and CD14- (fibroblast-rich fraction) cells. RNA sample concentrations were measured twice using the DeNovix DS-11 Spectrophotometer (DeNovix Inc., Wilmington, DE, USA) and adjusted to a concentration of 100 ng/*μ*l; a total of 1 *μ*g of RNA was used for cDNA synthesis. *NGF*, *CD14*, and *CD90* mRNA expression in CD14+ and CD14- cells isolated from 8 patients were separately examined using qPCR. *NGF* expression (*NGF*/*GAPDH*) in CD14+ and CD14- cells was determined using the delta-delta CT method. When the average *NGF* expression (*NGF*/*GAPDH*) level in CD14- cells was 1, the relative expression (CD14+ cells/CD14- cells) was calculated.

### 2.5. Cell Sorting

The CD14+ fraction from the synovium of hOA patients contains a heterogeneous population of CD14^high^ and CD14^low^ cells. We used synovial samples from 8 patients with hOA to examine *NGF* expression in CD14^high^ and CD14^low^ populations. To obtain synovial cells, each synovial sample was enzymatically digested, as described above. The synovial cells isolated from each patient were subsequently reacted with R-phycoerythrin- (PE-) conjugated anti-human CD14 (BioLegend) and fluorescein isothiocyanate- (FITC-) conjugated anti-human CD45 (BioLegend). After washing twice in PBS, CD14+ cells were sorted into CD14^high^ and CD14^low^ cells using the BD FACS Aria cell sorting system (Becton Dickinson, CA, USA). CD14^high^ and CD14^low^ cells were centrifuged at 300 g for 10 min and dissolved in TRIzol. RNA concentrations were measured twice using the DeNovix DS-11 Spectrophotometer (DeNovix Inc.) and adjusted to a concentration of 1 ng/*μ*l; a total of 10 ng of RNA was used for cDNA synthesis. After cDNA synthesis, as described above, a mixture comprising SuperMix, a reagent from Perfecta Preamp SuperMix (Quanta), 5 *μ*l primer pool (500 nM forward and 500 nM reverse), and 35 *μ*l cDNA was prepared to a final volume of 10 *μ*l for preamplification PCR. The program used was as follows: 3 min at 95°C, followed by 14 cycles of 15 s at 95°C and 3 min at 60°C. The resulting PCR products were diluted (1 : 16) and used in qPCR to examine *NGF* expression. *NGF* expression (*NGF*/*GAPDH*) was determined using the delta-delta CT method in CD14^low^ and CD14^high^ cells. When the average *NGF* expression (*NGF*/*GAPDH*) level in CD14^low^ cells was 1, the relative expression (CD14^high^ cells/CD14^low^ cells) was calculated.

### 2.6. Statistical analyses

Patients' demographic and clinical assessment data are presented as mean and standard deviation. Gene expression is expressed as median, percentiles, and minimum and maximum values. We performed power analysis with *α* = 0.05 and power = 0.80 using G∗POWER3 to calculate the optimal sample size for our study. The analysis indicated that 44 samples were needed to detect a correlation between *NGF* mRNA expression in synovial samples and clinical assessments. Thus, samples from 50 patients were used to investigate these correlations. Further, power analysis indicated that seven and eight samples were needed to detect a difference in *NGF* mRNA levels between CD14+ and CD14- cells and between CD14^high^ and CD14^low^ cells, respectively. Therefore, samples from 8 patients each were used to investigate these differences. Comparison of *NGF* mRNA expression level by radiographic hOA progression was investigated using the Mann-Whitney *U* test. Spearman's correlation coefficient was used to investigate the relationships between *NGF* mRNA expression in synovial tissue and CSI and JOA hip score. *CD14*, *CD90*, and *NGF* mRNA expression were compared between CD14+ and CD14- cell fractions using the Wilcoxon signed-rank test, as was *NGF* mRNA expression in each subset of CD14+ cells. All statistical comparisons were conducted using SPSS software (version 19.0, IBM, NY, USA). *P* < 0.05 was used to indicate statistical significance.

## 3. Results

### 3.1. Correlation between Synovial *NGF* Expression and Clinical Scores

**Figure 1:**
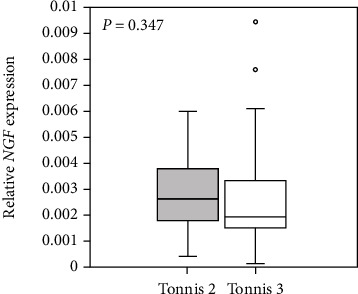
Synovial *NGF* mRNA expression in hip osteoarthritis patients with Tonnis grades 2 and 3. *P* < 0.05, statistically significant difference between patients with Tonnis grades 2 and 3 using the Mann-Whitney *U* test.

The 50 eligible patients (5 men and 45 women) provided consent to participate in the study (Table 2). The mean age was 64.0 ± 9.8 years, and all patients were diagnosed with advanced and end stages (Tonnis grade 2–3) of OA. There were no differences in *NGF* expression level between those with Tonnis grade 2 versus 3 (Figure 1).

**Figure 2:**
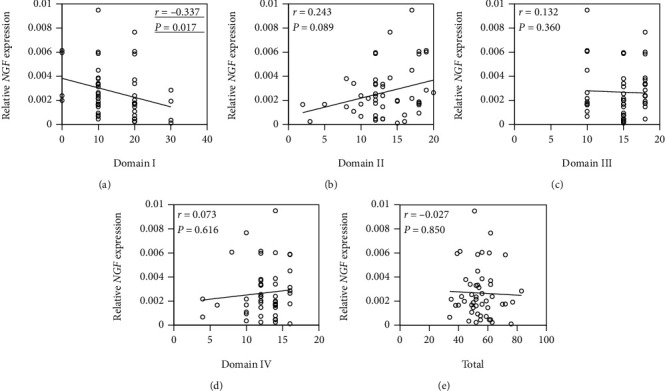
Correlation between *NGF* expression in synovial tissue and the Japanese Orthopaedic Association (JOA) score in patients with hip osteoarthritis. Correlation between *NGF* expression in synovial tissue and (a) domain I (pain), (b) domain II (range of motion), (c) domain III (gait), (d) domain IV (ability to perform activities of daily living), and (e) total JOA score. Data are Spearman's rho correlation coefficient. *P* < 0.05, statistically significant difference (underlined).

**Figure 3:**
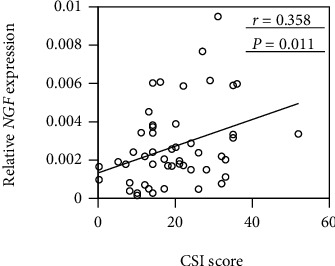
Correlation between *NGF* expression in synovial tissue and the Central Sensitization Inventory (CSI) score in patients with hip osteoarthritis. Data are Spearman's rho correlation coefficient. *P* < 0.05, statistically significant difference (underlined).

As shown in Figure 2, there was a negative correlation between JOA pain and *NGF* mRNA expression in synovial tissue (*r* = −0.337, *P* = 0.017). In contrast, there was no correlation between the 3 JOA domain scores or the total score and *NGF* mRNA expression (range of motion, *r* = −0.243, *P* = 0.089; gait, *r* = 0.132, *P* = 0.360; ability to perform activities of daily living, *r* = 0.073, *P* = 0.616; total, *r* = −0.027, *P* = 0.850). The CSI score was positively correlated with synovial *NGF* mRNA expression level in patients with hOA (*r* = 0.358, *P* = 0.011; Figure 3).

### 3.2. Expression of *NGF* in Synovial Macrophages and Fibroblasts

**Figure 4:**
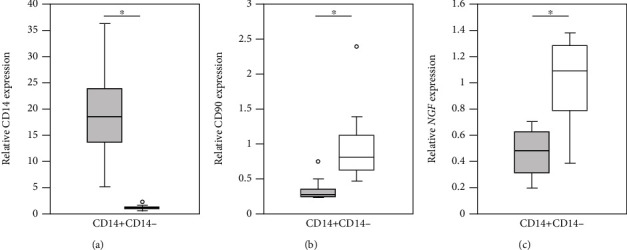
*NGF*, *CD14*, and *CD90* mRNA expression in CD14+ and CD14- synovial cells extracted from the synovial tissue of hip osteoarthritis patients. Comparison of (a) *CD14*, (b) *CD90*, and (c) *NGF* mRNA expression in CD14+ and CD14- cells (*n* = 8). ^∗^*P* < 0.05, statistically significant difference between CD14+ and CD14- cells using the Wilcoxon signed-rank test.

Samples from 8 patients (2 men, 6 women) aged 65.5 ± 7.2 years with radiographic hOA (Tonnis grade 2–3) were used to examine *NGF* mRNA expression in synovial macrophages and fibroblasts. To identify the cell population in CD14+ and CD14- fractions, we performed qPCR analysis of *CD14* (macrophages) and *CD90* (fibroblasts). The analysis showed that *CD14* mRNA levels in CD14+ cells were significantly increased compared to those in CD14- cells (*P* = 0.012; Figure 4(a)). In contrast, *CD90* expression was significantly elevated in CD14- compared to CD14+ cells (*P* = 0.017; Figure 4(b)). This indicates that CD14+ and CD14- fractions contained macrophages and fibroblasts, respectively. *NGF* mRNA expression was significantly elevated in CD14- compared to CD14+ cells (*P* = 0.036; Figure 4(c)).

### 3.3. *NGF* Expression in Synovial CD14+ Cell Subsets from hOA Patients

**Figure 5:**
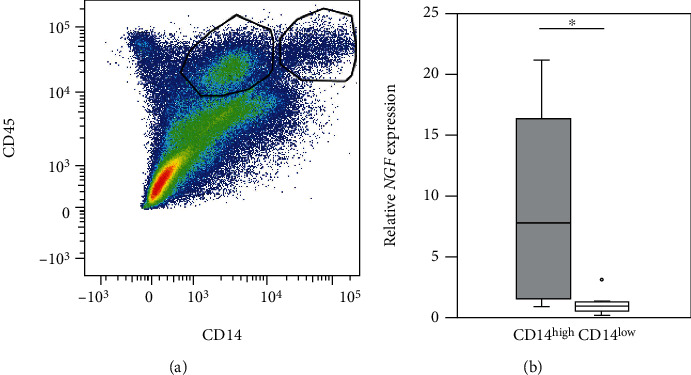
*NGF* mRNA expression in CD14+ synovial cells, a heterogeneous population of CD14^high^ and CD14^low^ cells, from hip osteoarthritis patients. (a) Dot plot showing CD14+ cells among hOA synovial cells. *x*-axis, CD45; *y*-axis, CD14. (b) *NGF* mRNA expression in CD14^high^ and CD14^low^ cells among CD14+ cells (*n* = 8). ^∗^*P* < 0.05, statistically significant difference between CD14^high^ and CD14^low^ cells using the Wilcoxon signed-rank test.

Cell sorting analysis was performed on samples from 8 patients (all women) aged 66.6 ± 8.1 years with hOA (Tonnis grade 2–3) to identify the characteristics of CD14+ cell subsets. As shown in Figure 5(a), CD14+ cells gated with CD45 comprised a heterogeneous population of CD14^high^ and CD14^low^ cells. CD14^high^ cells expressed higher levels of *NGF* mRNA than CD14^low^ cells in the synovium of hOA patients (*P* = 0.008; Figure 5(b)).

## 4. Discussion

We showed that synovial *NGF* mRNA expression was negatively and positively correlated with JOA pain and CSI scores, respectively. Both CD14+ and CD14- synovial cells extracted from hOA patients expressed *NGF*, with CD14- cells showing higher *NGF* expression than CD14+ cells. Further, we found that CD14+ synovial cells from hOA patients comprised a heterogeneous population of CD14^high^ and CD14^low^ cells. CD14^high^ cells expressed significantly higher levels of *NGF* mRNA than CD14^low^ cells. These findings suggest that elevated *NGF* levels in the synovium of hOA patients may be associated with pain through CS. In addition to synovial fibroblasts, macrophages, particularly CD14^high^ macrophages, may also mediate *NGF* expression in patients with hOA.

A previous report suggested that the binding of NGF to TrkA receptors on the peripheral terminals of nociceptors and the surface of immune cells may directly contribute to acute peripheral sensitization [19]. NGF is thought to contribute indirectly to CS via its downstream influence on transcription [7, 20]. Lewin et al. showed that systemic administration of NGF to a rat model caused mechanical and thermal hyperalgesia [7]. Moreover, Ashraf et al. reported that intraarticular injection of NGF in a rat OA model lowered the hind paw mechanical withdrawal threshold [21]. In addition, Sagar et al. demonstrated that intraarticular injection of NGF contributed to spinal nociceptive sensitization in a rat OA model [22]. However, whether or not these mechanisms are also present in humans is unclear. Our findings indicate that there is a significant correlation between *NGF* mRNA expression levels in the synovium and JOA pain and CSI scores in hOA patients. There were no differences in *NGF* expression between patients with radiographic hOA Tonnis grade 2 versus 3. Therefore, synovial NGF expression may contribute to CS and pain in human hOA patients.

Evidence indicating that NGF expression is upregulated in synovial fibroblasts from kOA patients suggests that NGF is a key contributor to pain pathology in OA patients [8, 9, 23]. An immunohistochemical study showed that NGF is predominantly expressed in fibroblasts compared to macrophages in the synovium [11]. We found that *NGF* mRNA was expressed in both CD14+ and CD14- synovial cells from hOA patients and that *CD90* and *NGF* were significantly elevated in CD14- compared to CD14+ synovial cells suggesting that NGF may be mainly expressed in synovial fibroblasts in patients with hOA.

Accumulating evidence suggests that synovial macrophages may contribute to pain [24]. In this study, we investigated a possible role for NGF expression in the regulation of macrophage-associated pain in hOA. While CD14+ cells expressed lower levels of *NGF* than CD14- cells, expression was significantly elevated in CD14^high^ cells compared to CD14^low^ cells among synovial CD14+ cells from hOA patients. CD14 concentration in synovial fluid has been shown to be positively correlated with the severity of self-reported knee pain [25]. CD14^high^ subsets are thus thought to expand under arthritic conditions [26]. Taken together, our findings and those of previous studies suggest that synovial expression of *NGF* in CD14^high^ macrophages may contribute to pain in hOA patients.

We showed that NGF expression levels differed between CD14^high^ and CD14^low^ macrophages. However, because it was difficult to distinguish between CD14^high^ and CD14^low^ cells using immunohistochemical procedures, NGF expression and localization of these cells in the synovium remain unclear. Identification of other markers that can be used to distinguish these cells using immunohistochemistry is needed.

## 5. Conclusions

Synovial NGF expression correlates with the degree of pain and CS in hOA patients. NGF is predominantly expressed in synovial fibroblasts. Among synovial macrophages, NGF is highly expressed in CD14^high^ cells. Our results suggest that NGF-expressing cells may be a novel target for therapeutic strategies against chronic pain with CS in hOA.

## Figures and Tables

**Table 1:** Sequences of the primers used in this study.

Primer	Sequence (5′–3′)	Product size (bp)
NGF-F	CCCATCCCATCTTCCACAGG	74
NGF-R	GGTGGTCTTATCCCCAACCC	
CD14-F	TCCCTCAATCTGTCGTTCGC	150
CD14-R	ATTCCCGTCCAGTGTCAGGT	
CD90-F	GACCCGTGAGACAAAGAAGC	138
CD90-R	CCCTCGTCCTTGCTAGTGAA	
GAPDH-F	TGTTGCCATCAATGACCCCTT	202
GAPDH-R	CTCCACGACGTACTCAGCG	

NGF: nerve growth factor; GAPDH: glyceraldehyde-3-phosphate dehydrogenase; bp: base pairs.

**Table 2:** Patients' demographics and clinical assessments data (*N* = 50).

hOA grade	Tonnis 2	Tonnis 3	*P* value
Sex, male/female, *N*	2/11	3/34	0.452
Age (years)	64.4 ± 11.2	64.4 ± 9.8	0.965
JOA score			
Pain (0-40 points)	16.1 ± 8.7	13.8 ± 7.2	0.366
Range of motion (0-20 points)	14.8 ± 3.8	12.8 ± 4.1	0.207
Gait ability (0-20 points)	15.8 ± 2.3	14.5 ± 3.2	0.281
Ability to perform activities of daily living (0-20 points)	13.1 ± 2.1	12.4 ± 3.0	0.739
Total (0-100 points)	58.2 ± 12.1	53.5 ± 9.9	0.268
CSI score (0-100 points)	20.8 ± 8.8	19.6 ± 11.2	0.740

All data are reported as mean ± standard deviation ratings, unless otherwise indicated. Continuous variables were calculated using the Mann-Whitney *U* test, and categorical variables were calculated using the chi-square test. *P* values < 0.05 were considered to indicate statistical significance. hOA: hip osteoarthritis; JOA: Japanese Orthopaedic Association; CSI: Central Sensitization Inventory.

## Data Availability

The datasets supporting the conclusions of this article are included within the article. The raw data can be requested from the corresponding author.
